# Mutation divergence over space in tumour expansion

**DOI:** 10.1098/rsif.2023.0542

**Published:** 2023-11-22

**Authors:** Haiyang Li, Zixuan Yang, Fengyu Tu, Lijuan Deng, Yuqing Han, Xing Fu, Long Wang, Di Gu, Benjamin Werner, Weini Huang

**Affiliations:** ^1^ Group of Theoretical Biology, The State Key Laboratory of Bio-control, School of Life Sciences, Sun Yat-sen University, Guangzhou 510275, People’s Republic of China; ^2^ Evolutionary Dynamics Group, Centre for Cancer Genomics and Computational Biology, Barts Cancer Institute, Queen Mary University of London, London, UK; ^3^ The first affiliated hospital of Guangzhou Medical University, Guangzhou, People’s Republic of China; ^4^ School of Mathematical Sciences, Queen Mary University of London, London, UK

**Keywords:** spatial tumour heterogeneity, spatial sampling, agent-based model, tumour evolution, mutation divergence

## Abstract

Mutation accumulation in tumour evolution is one major cause of intra-tumour heterogeneity (ITH), which often leads to drug resistance during treatment. Previous studies with multi-region sequencing have shown that mutation divergence among samples within the patient is common, and the importance of spatial sampling to obtain a complete picture in tumour measurements. However, quantitative comparisons of the relationship between mutation heterogeneity and tumour expansion modes, sampling distances as well as the sampling methods are still few. Here, we investigate how mutations diverge over space by varying the sampling distance and tumour expansion modes using individual-based simulations. We measure ITH by the Jaccard index between samples and quantify how ITH increases with sampling distance, the pattern of which holds in various sampling methods and sizes. We also compare the inferred mutation rates based on the distributions of variant allele frequencies under different tumour expansion modes and sampling sizes. In exponentially fast expanding tumours, a mutation rate can always be inferred for any sampling size. However, the accuracy compared with the true value decreases when the sampling size decreases, where small sampling sizes result in a high estimate of the mutation rate. In addition, such an inference becomes unreliable when the tumour expansion is slow, such as in surface growth.

## 1. Introduction

The accumulation of somatic mutations is a natural consequence of cell turnover during tissue formation and maintenance [1–3]. During cell divisions, genetic variants such as single-nucleotide polymorphisms (SNPs) and copy number variations can happen, which may lead to fitness differentials between cells and eventually tumour initiation [4,5]. While only a small subset of mutations will be driver events and confer fitness advantages, neutral passenger mutations continuously accumulate and increase intra-tumour heterogeneity (ITH) [6–8]. Tumours with high genetic heterogeneity are considered more difficult to treat and with higher recurrence rates in clinical practice [7–10]. This may be for example due to the higher chance of pre-existing resistance before in treatment in tumours with high ITH [11–16].

The patterns of ITH are driven by both spatial and temporal dynamics [17,18]. While temporal samples are often infeasible, the distribution of accumulated mutations has been used to reveal the evolutionary history of tumours [19]. Sottoriva *et al.* [20] introduced a ‘Big Bang’ model, where neutral passenger mutations can accumulate fast in the early expansion of tumours after tumorigenesis. The distribution of those neutral mutations follows a power law decay along the increase of mutation frequency by theoretical predictions [21,22], which is consistent with around one-third of tumour samples across many cancer types in The Cancer Genome Atlas (TCGA) database [23]. In large populations, new driver mutations can arise and even lead to clonal competitions in theory [24] as well as in experimental observations [25], which immediately increases the complexity of revealing tumour history and parameters through ITH pattern in bulk samples [26]. Spatial studies with multi-region sequencing have been carried out to compensate the practical constraints of obtaining temporal samples in solid tumours [17,18,27–32]. Different spatial models of cancer evolution are further developed to analyse the spatial impact on ITH, as well as to use spatial information, e.g. from multi-region genomic data to infer evolutionary parameters such as mutation rates and selection strength [31,33–37].

Using a three-dimensional lattice, Waclaw *et al.* [33] modelled exponentially expanding tumours with continuously arising mutations with and without dispersal. They showed that the ITH level and selective advantage of tumour cells have a negative correlation where tumours without driver mutations are more genetically heterogeneous. In addition, high heterogeneity leads to the rapid onset of resistance to chemotherapy, and a small amount of localized cellular dispersal can cause a faster growth of tumours formed of conglomerates of balls with more similar genetic alterations, compared with spherical tumours without cell dispersal. Ryser *et al.* [31] investigated the role of early tumour cell migration in shaping the patterns of private mutation in colorectal tumours through an extension of the ‘Big Bang model’ [20]. Their results showed that detecting the same private mutations in opposite sides of the final tumours indicates early cell mixing and movement, and that neutral evolution leads to high local ITH under the single expansion hypothesis of tumorigenesis. Chkhaidze *et al.* [34] modelled the spatial constraints of tumour growth through a pushing parameter, and showed that the phylogeny of multiple samples deviates more from the ground truth under stronger spatial constraints due to higher sampling bias. Combining theoretical and clinical data, Lenos *et al.* [38] and Fu *et al.* [39] also visualized the heterogeneity distribution and clonal diversity under volume and surface growth, where cells divide evenly throughout the whole tumour in volume growth but only in the boundary with the empty space in surface growth. Lenos *et al.* [38] quantitatively analysed the lineage tracing data from primary colon cancer and found that the stem cells driving tumour growth are mostly at tumour edges, and stem cell properties change over time depending on the cell location. Fu *et al.* [39] showed that the complete variability of clone size is due to spatio-temporal regulation of necrosis and that surface growth had a greater effect on sub-clonal diversity than volume growth. They also revealed that clonal diversity decreased sharply with the growth time in the surface model. More recently, Noble *et al.* [36] compared the tumour dynamics under different spatial structures in computational models, which reflect tissue architectures ranging from non-spatial as leukaemia, various gland structures as colorectal and breast cancer, to surface growth in certain types of liver cancer. While no spatial constraints result in rapid clonal expansion of cells carrying driver mutations, strong spatial constraint like surface growth will suppress selection and lead to tumour patterns similar to those under neutral evolution. Beside of lattice models, Gallaher *et al.* [40] applied the off-lattice model to systematically study and predict the spatial dynamic evolution of heterogeneous tumours in different treatment periods. Their results showed that evolution-based strategies by exploiting the cost of resistance can delay treatment failure, and fewer drugs and more vacation-oriented treatment can reduce tumour heterogeneity.

Beyond competition and constraints on pure physical space, the micro- and macro-environment play a significant role in tumour development especially on the non-genetic factors [41]. Anderson *et al.* [42] introduced hybrid models to simulate tumours in context, where the tumour environment such as extracellular matrix dynamics and oxygen or nutrient concentration are governed by partial differential equations, and cell reproduction and death are modelled in two-dimensional lattice with feedbacks between the two scales. Gerlee & Anderson [43] extended the original hybrid model and included mutations to investigate the impact of micro-environment on tumour clonal evolution. They observed more aggressive tumour phenotypes and a higher ITH in harsher environment with a low oxygen concentration. Hybrid models have been further developed to study for example tumour stroma interactions on prostate cancer [44], hierarchical and stem-cell driven tumour dynamics [45] and metastases [46]. More recently, Gallaher *et al.* [47] used a hybrid model to study phenotype heterogeneity in Glioblastoma, where a cell’s phenotype is an outcome of its inheritable traits and the influence of the environment modelled as a continuous field of platelet-derived growth factor distribution. Their results showed that both environmental and intrinsic factors are required to best fit the observed phenotypic heterogeneity and cell migration patterns in rat glioma data. While tumour–environment interactions most likely will impact on the genetic heterogeneity as well, here we focus on a simpler scenario and quantify how the physical space constraint and sampling distance will impact on the measured genetic heterogeneity between samples. While our conclusions are based on pure spatial competition, deviation from the mutation divergence patterns observed in our model can reflect other major driving mechanisms beyond space limitation, where hybrid models can be used to further extend our study.

While different growth modes of solid tumours are related to the strength of spatial constraints and likely to be tissue-specific [48], we simplify this as a continuous inter-cellular push rate (0 ≤ *p* ≤ 1) similar to Sottoriva *et al.* [49], Ryser *et al.* [31], Chkhaidze *et al.* [34] in an agent-based model in two- or three-dimensional lattice. Different values of the push rate, i.e. the likelihood of a cell pushing neighbour cells outward when there are no direct empty nodes around it in division, refer to different modes of tumour growth including the surface (*p* = 0) and exponential growth (*p* = 1) as two boundary examples.

More and more experimental and theoretical studies have demonstrated the importance of sampling itself on the interpretation of measured ITH. Opasic *et al.* [50] investigated how sampling will impact on the accuracy of identifying clonal mutations in a lattice model and showed non-random sampling from a circular spatial pattern might improve the classification of true clonal mutations compared with random sampling. Ling *et al.* [51] evaluated the spatial distribution of point mutations, by sequencing approximately 300 sampled regions within the circular range of a single tumour, and found that cells sampled farther away from the sample centre carry more mutations. Experimental work from Masugi *et al.* [52] and Zhao *et al.* [53] indicated that cell density and metastatic likelihood differ between tumour centre and margin. Quantitative measures of how sampling can impact on the ITH will provide theoretical foundations in how to implement multi-region sampling in practice in future. Here, we take random samples with various sizes in each simulation and analysis the average pattern of ITH between samples. In comparison, we also take multiple samples from the central ((2/3)*R*) and marginal regions ((1/3)*R*) of simulated tumours with radius *R*. We record the accumulation of point mutations, compare mutation frequencies above a detection limit and infer mutation rates in each sample. To compare the ITH between samples, we apply the Jaccard index to quantify the mutation composition difference, which has been used to quantify mutation diversity in much genetic data [54–59]. The more mutations shared between two samples, the higher the Jaccard index is and the lower the ITH is.

Our results show that under a given tumour expansion mode (fixed push rate), the ITH increases with the sampling distance, which is consistent with observations in experimental data [51,60]. This pattern holds for various sampling sizes, for random, centre and margin sampling, and for two- or three-dimensional simulations. The faster a tumour expands (larger push rates), the slower the ITH increases along the sampling distance. In addition, we construct the distributions of variant allele frequencies (VAF) and infer the mutation rate for each sample. When push rates are large such as in exponentially expanding tumours, a linear relation is always observed between the number of mutations and accumulative mutation frequencies independent of sampling sizes. Using this linear relation, we can estimate the mutation rates and find that the sampling size strongly impacts the inferred mutation rates. Smaller sampling sizes lead to an overestimate of inferred mutation rates as well as a larger variance among samples. Increasing the sampling size improves the inference with a more accurate mean compared with the true value and smaller variance among samples. When the tumour expansion is slower such as in surface growth, such inference becomes impossible because the linear relationship does not hold. The smaller the sampling size, the larger the VAF deviates from a linear relation.

## 2. Methods

### 2.1. Stochastic simulations of tumour growth in space and mutation accumulation

**Figure 1.**
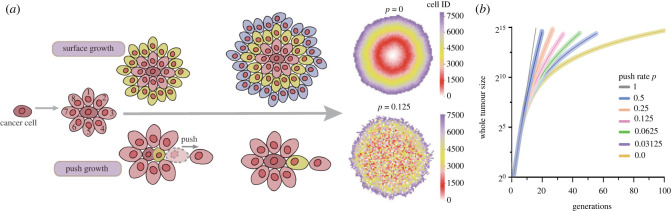
The spatial growth under different push rates. (*a*) when push rate *p* = 0, it refers to the surface growth, where only cells on the surface have an empty spot in its direct neighbours and can divide. For other values of the push rate, cells not on the surface can also divide by creating an empty spot among its neighbours through pushing. We allocated a unique ID for each new cell. The larger the cell ID, the later the cell was reproduced. (*b*) To reach the same tumour size, surface growth takes more generations of reproductions compared with exponential growth under *p* = 1. While mutation accumulation only happens in cell divisions in our model, for tumours of the same size, the push rate will impact on the mutation burden in tumours and also the spatial distribution of those mutations. The shadowed area is 100 simulations under the corresponding push rate, where their averages are shown as solid lines. (Figure 1*b* is 100 times simulation, cell number is around 2^15^, *p* = 1 grow to 2^15^ cells only 15 generations. When *p* = 0 divide 100 generations can grow approx. 27 500 cells.)

We use an agent-based model to simulate tumour growth [33,34,42,49,61–63] and perform the majority of our simulations in two-dimensional lattice where each cell has eight direct neighbours. Tumours develop from a single cell, which is seeded in the centre of the lattice. When a cell divides into two daughter cells, one cell remains in its original location (*L*_*p*_), and the other daughter cell is located in a randomly selected empty space among its direct neighbours. If there is no free space, with a probability *p* (0 ≤ *p* ≤ 1), a new space can be created by pushing a randomly selected neighbour cell (at location *L*_*d*_) and all the rest of the cells along the direction of *L*_*p*_ and *L*_*d*_ outwards for one position until an empty space was reached [34]. For two extreme values of the push rate, *p* = 0 refers to the surface growth where only cells in the outskirt of the tumour would divide and *p* = 1 refers to an exponential growth where all cells divide in each time step (figure 1). In a comparison, we implement a similar pushing algorithm as in [33,63], where instead of pushing a random neighbour, the nearest empty lattice point is first searched and the cells between the dividing cell and this empty point are pushed along the shortest path. To reduce our computational cost, we also performed the same agent-based model with pushing a random neighbour in three-dimensional lattice in a few parameter sets and compared the ITH patterns from two- and three-dimensional simulations.

In each cell division, random point mutations can happen in both daughter cells compared with the parent cell. The number of new mutations in each daughter cell follows a Poisson distribution [6,23,26,33,64], where *λ* is the mean value representing the average mutations (mutation rate) per cell division. Thus, the probability to have *k* new mutations in one daughter cell is *λ*^*k*^ e^−*λ*^/*k*!. If not specified, we use *λ* = 10, which is conventionally considered as the average number of point mutations per cell division in tumours [26,65,66].

### 2.2. Spatial sampling

To understand how mutations spread over space, we test two different spatial sampling methods, i.e. random sampling and centre-margin sampling. For the random sampling method, we sample 500 locations randomly in each simulated tumour and collect cells in a rectangular area around these locations (figure S1a in electronic supplementary material). To investigate the impact of sampling sizes on our diversity measures, we vary sample size from 100 to 3600 cells (around 0.6–21.6% of the whole tumour). Alternatively, we divide simulated tumours into the central region (a circle of two-thirds of the radius from the tumour centre) and the marginal region (the rest one-third ring structure). We randomly sample 500 rectangular areas with 100 cells in each sample (around 0.6% of the whole tumour) in the margin and 500 samples with 100 cells in the centre region (figure S1b in electronic supplementary material).

### 2.3. Measurements of intra-tumour heterogeneity between samples

We use a statistical measurement, the Jaccard index, to compare the diversity between samples. Supposing *A* and *B* are the set of mutations in two samples, the similarity of mutations between the two samples is given by

2.1 $$J(A,B)=\frac{\left|A\cap B\right|}{\left|A\cup B\right|}=\frac{\left|A\cap B\right|}{\left|A|+|B|-|A\cap B\right|},$$

where A∩B is the intersection of mutations between these two samples and A∪B is their union. Thus, by definition the Jaccard index will be between 0 and 1. By using the Jaccard index, we can quantify how similar mutations are accumulated in samples over space. More specifically, we quantify how the Jaccard index is related to the sampling distance, which is measured by the Euclidean distance between the centres of samples.

### 2.4. Mutation rate inference and Kolmogorov–Smirnov test

Another classical measurement for patterns of mutation accumulation in population genetics is the frequency distribution of mutations among tumour cells in each sample, which is called variant allele frequency (VAF) distribution in cancer research. We are interested in how different growth dynamics, such as push rates as well as spatial sampling, impact on the measured VAF distributions. For simplicity, we simulate only one driver event where genetic changes lead to the initiation of a single tumour cell, which seeds for the tumour growth and the accumulation of random neutral mutations during the tumour expansion. In this scenario, the accumulated mutations follow a theoretical expectation of power-law decay, where the number of mutations of a given frequency decrease along with the mutation frequency *f* [23]. The cumulative distribution is a linear relation as *M*(*f*) = (*μ*/*β*)((1/*f*) − (1/*f*_max_)), and the slope could determine the effective mutation rate *μ*/*β* (the true mutation rate scaled by death). We constructed the cumulative VAFs for all samples and compared how the push rates, sampling methods and sizes would impact on this measurement. We quantify how much the observed cumulative agrees with a linear regression by Kolmogorov–Smirnov (KS) test [67], which measures the maximum distance of the linear regression with the observed cumulative VAF curve. If the observed cumulative VAFs agree with linear regression, we infer the mutation rates using the equation above.

## 3. Results

### 3.1. Spatial mixing increases and variance of mutation frequencies decreases with push rates

**Figure 2.**
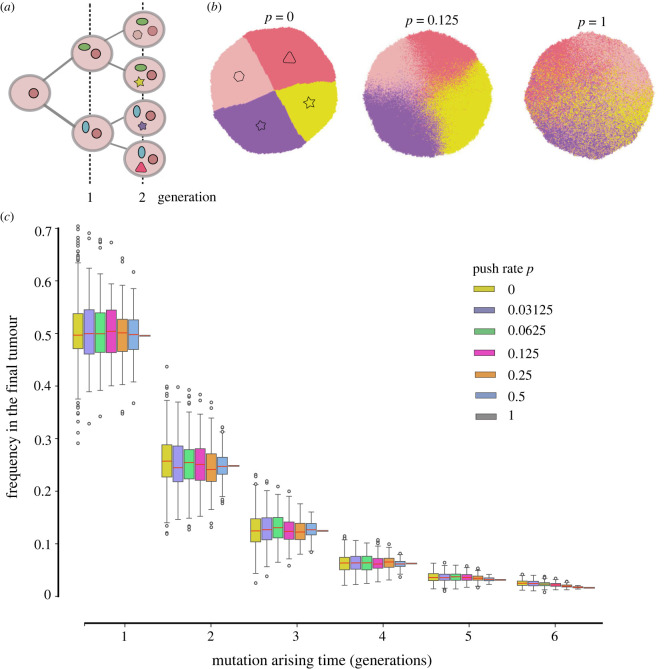
The impact of push rates on distributions of early mutations. (*a*) We record the identities of early mutations, including the mutation ID and the time when they were first present in the population. (*b*) One example of the spatial distribution of unique mutations from four cells after the second round of cell division (the distribution of four types of mutation in the second generation, cell number is approx. 2^14^). (*c*) Frequencies of all early mutations in growing tumours. These mutations arise at different rounds of early cell divisions (300 times simulation, cell number is approx. 2^14^, cut last three points). While the push rates change, thus the spatial distributions of those mutations differ as in (*b*); their final frequencies are 0.5, 0.25, 0.125, 0.0625 and 0.03125 with the push rate. Here, the red lines in colourful bars are the average mean value of frequencies of all mutations from 300 times simulation realizations, and the bars are the variance. (*λ* = 10, the final tumour size is 2^14^.)

When the push rate is low (*p* = 0), cells grow slowly and mainly on the surface. From spatial patterns constructed by the cell ID (figure 1*a*), where the cell born later is assigned a larger ID number, we observed clear circular boundaries among early and later born cells. However, when the push rate increases, these spatial boundaries become loose. Instead, spatial mixing among early and later born cells appears. Similar effects are observed in the spatial pattern of mutations. In figure 2*b*, we demonstrate the spatial pattern of four different mutations randomly picked up from four cells born in the second generation. When *p* = 0, clear boundaries among cells carrying those mutations exist. With the increase in push rate, cells are carrying different mutations mix in space.

The earlier a mutation arises during the tumour growth, the higher frequency it reaches in the final tumour (figure 2*c*). This pattern is consistent under all push rates. Arising at the same tumour generation, the mean value of the frequency these mutations can reach in the final tumour is independent of the push rates. However, the variance increases monotonically when the push rate decreases.

### 3.2. The growth modes have a strong impact on the variant allele frequencies distribution and mutation rate inference

**Figure 3.**
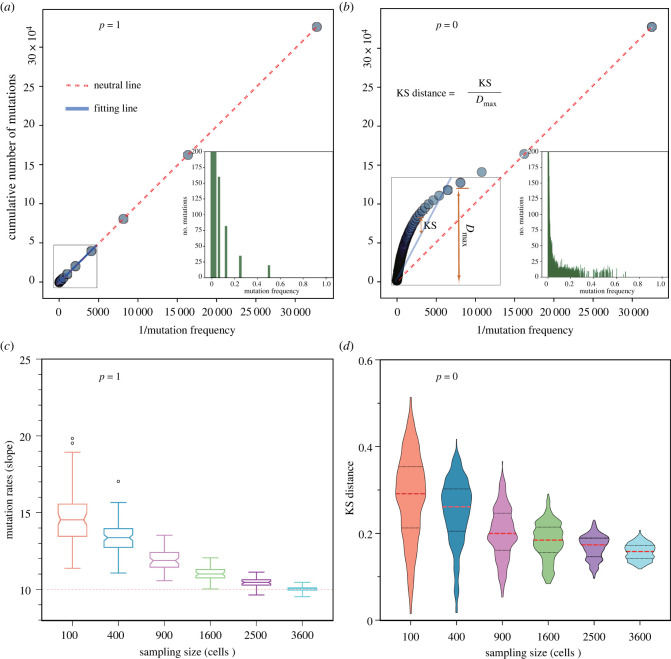
Mutation accumulations under different pushing rates and sampling sizes. (*a*) Cumulative distribution of mutations when *p* = 1, where the computational simulations agree with the linear expectation (red dashed lines) of neutral mutations accumulated in exponentially expanding tumours. (*b*) As the push rate decreases, the tumour growth is closer to the surface growth (*p* = 0). The corresponding cumulative distributions of mutations deviate from the linear expectation, which is measured by the Kolmogorov–Smirnov (KS) distance. (*c*) Under exponential growth (*p* = 1), sampling does not change the pattern of linear regression fitting for accumulated mutations. However, the estimated mutation rate increases when the sampling size decreases. (*d*) Under surface growth (*p* = 0), the deviation from the linear expectation increases when the sampling size is smaller. (*λ* = 10, the final tumour size is 2^14^).

Next, we construct the VAF of all mutations accumulated through tumour growth. Mutations with the frequency of less than 0.01 in the final tumour are discarded, as in reality, it is hard to detect such lower frequency mutations in a standard sequencing depth. Figure 3*a*,*b* shows examples of single simulations under two boundaries of push rates, *p* = 0 and *p* = 1. Without mimicking sequencing noise in our simulation, when push rate *p* = 1, the VAF distribution is discrete with mutations at frequencies 0.5, 0.25, 0.125, 0.0625 and so on (figure 3 inset). The cumulative VAF distribution is a linear line and perfectly agrees with the theoretical expectation under neutral selection. On the contrary, when only surface growth is allowed under *p* = 0, there is a wide and relatively continuous VAF distribution covering all intermediate frequencies and even with few mutations exceeding 0.5 (figure 3*b* inset), which is the frequency of most clonal mutations in diploid populations. The cumulative VAF distribution under surface growth strongly deviates from a linear relation, which is quantified by the KS distance. The larger the KS distance, the further away the cumulative VAF distribution deviates from a linear regression.

We measure the KS distance for different push rates and sampling sizes under random sampling. Note, to eliminate the extremely low-frequency mutations (less than 0.01), we discard the last few dots in the cumulative VAF distributions for the linear regression (e.g. the last three dots in figure 3*a*,*b*). We found that the push rates have a strong impact on this measurement, where the KS distance keeps a relatively high level when push rates are small (figure 3*d* and figure S4*a–c* in electronic supplementary material). This means that mutation rate inferences based on a linear regression are not reliable under small *p*. The KS distance decreases when *p* becomes larger (figure S4*d*,*e* in electronic supplementary material), and a linear regression is reasonable across all sampling sizes (figure S2*d*,*e* in electronic supplementary material). Thus, we can infer the mutation rates based on the slope of the linear regression under large *p*.

Figure 3*c* shows the fitting results of 100 simulations with 500 random sampling each under *p* = 1. While increasing the sampling size, the inferred mutation rate is closer to the true value with reduced variance. On the contrary, although we also observe a close-to-linear relation under small *p* (figure S2*a* in electronic supplementary material), the inferred mutation rate is often an overestimate compared with the true value (figure 3*c*, dashed line). In addition, the smaller the sampling size is, the large the variance of the mutation rate is.

### 3.3. Intra-tumour heterogeneity increases with the sampling distance

**Figure 4.**
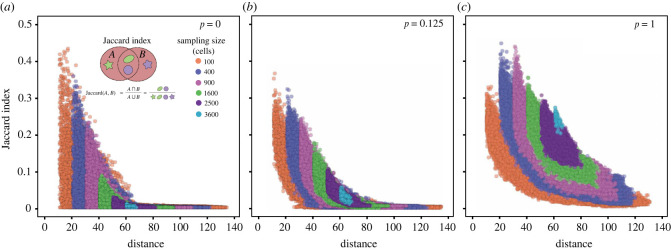
The mutation divergence over space. Pairwise comparison of mutation divergences between samples are measured by Jaccard index. As the sampling distance increases, fewer mutations are shared between the samples, and the smaller the Jaccard index is. (*a*) *p* = 0. (*b*) *p* = 0.125. (*c*) *p* = 1. This holds for any push rates and any sampling size. For the surface growth (*a*), the Jaccard index decreases faster to 0 when the sampling distance increases compared with exponential growth (*c*). This effect is stronger when the sampling size is smaller (e.g. orange dots compared with purple dots). (1-time simulation, sampling 500 points for each sample size, cell number is approx. 2^14^, cut last three points.)

In each simulation, we first randomly sample 500 areas with various sampling sizes. We measure the ITH between spatially non-overlapping samples and quantify how this heterogeneity changes with spatial distances. We compare the samples pairwise to calculate the Jaccard index, which is inversely proportional to intra-tumour heterogeneity. Meanwhile, the spatial distances between samples are defined by the Euclidean distance between the central points of each sample. For various sampling sizes and push rates, the Jaccard index between two random samples decreases, thus the ITH increases, monotonically with the spatial distances (figure 4, figure S5 in electronic supplementary material). When the push rate *p* = 0 (surface growth), the Jaccard index drops down to 0 very fast (figure 4*a*), where the non-overlapping samples have fewer and fewer shared mutations when the sampling distance increases. When *p* increases, given the same sampling size and distance, the Jaccard index increases. This agrees with the observation of spatial mixing of cells carrying different mutations. When *p* = 1, cell spatial mixing reaches the highest level, and we seldom observe any Jaccard index as 0 even under the smallest sampling size and largest sampling distance, and there are always shared mutations among those samples. While the results in figure 4 are based on single simulations, 249 500 Jaccard indexes are calculated after the pairwise combination of 500 samples in a single simulation. Such a large number gives a stable pattern, which is consistent with results over 100 simulations (figure S6 in electronic supplementary material). In addition, an alternative pushing algorithm with the shortest path to the nearest empty spot gives qualitatively similar results (figure S8 in electronic supplementary material). However, in this pushing algorithm, compared with the dividing cell the nearest empty spot in a lattice is more likely to be at the same side of the tumour, which leads to more clustering of cells carrying the same genetic mutations, compared with the case when pushing happens in a random direction. Consequently, the Jaccard index in this alternative pushing algorithm is lower under the same parameter set (figure S8 in electronic supplementary material).

To understand the impact of sampling methods, we divided the simulated tumours into the central region (2/3 *R* circle) and margin region (1/3 *R* ring width), where *R* is the tumour radius (figure S1*b* in electronic supplementary material). We randomly sampled 500 areas (100 cells, 196 cells, 400 cells) in the margin and centre, respectively (figure S7 in electronic supplementary material). Then, we compared the relationships of the Jaccard index and spatial distance between samples in the margin and the centre region under the different push rates. The patterns are very similar to those observed in completely random sampling. The Jaccard index decreases with the increase of the sampling distance, and the push rates lead to a higher Jaccard index under the same sampling size. In addition, we see the Jaccard index is slightly higher between samples in the central region compared with the margin region. This is more obvious when push rates are small, where the spatial constraint is stronger and thus less mutations are shared between spatially non-overlapping samples. In summary, the two sampling methods do not alter the pattern of how ITH increases with the sampling distance qualitatively with increasing *p* (less spatial constraints). However, there is a small quantitative difference if we sample in the margin or centre of tumours.

### 3.4. Patterns of mutant divergence remain similarly between two- and three-dimensional models

**Figure 5.**
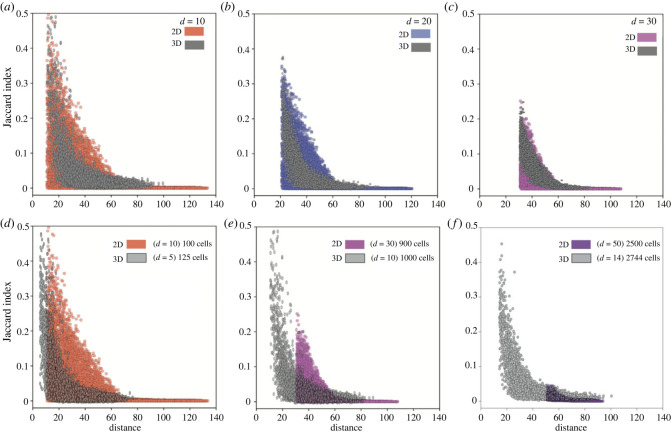
Comparison between the Jaccard index distribution between two- and three-dimensional simulations. Here, *d* refers to the diameter of a sample, where for the same value of *d*, there are more tumour cells in the samples in three-dimensional simulations compared with two-dimensional simulations. Meanwhile, the diameter of a sample is also the shortest sampling distance of two non-overlapping samples. (*a*–*c*) Compare the Jaccard distribution under the same sampling diameters between two- and three-dimensional simulations. (*d*–*f*) Compare the Jaccard distribution under a similar sampling size between two- and three-dimensional simulations. (1-time simulation under *p* = 0, sampling 500 points for each sample diameter or size, cell number is approx. 214 in the two-dimensional simulation and 105 in the three-dimensional simulation, cut last three points) were compared with the same sampling distance and the same number of sampling cells, respectively.

The majority of our simulations are based on two-dimensional lattice to reduce the computational cost of simulating tumours with large sizes and sampling distances, which results in a limitation as solid tumours are often three-dimensional. To explore this, we extend our agent-based model from two to three dimensions in some parameter sets (figure S9 in electronic supplementary material). We model the tumour growth up to 10^5^ cells in three-dimensional lattice and compare the Jaccard index between two- and three-dimensional simulations. In general, we observe a qualitatively similar pattern of Jaccard index over sampling distance and sampling sizes in three-dimensional compared with two-dimensional models. Interestingly, the diameter of a sample, which is also the shortest sampling distance of non-overlapping samples, determines a quantitatively comparable pattern of the Jaccard index (figure 5*a*–*c*). For a similar sampling size under two- and three-dimensional simulations, the Jaccard index is in general higher in two-dimensional compared with three-dimensional simulations even under the same sampling distance (figure 5*d*–*f*). This might be due to the fact that it takes more cell divisions to reach the same physical distance in three-dimensional compared with two-dimensional simulations, thus higher intra-tumour heterogeneity between samples.

### 3.5. Pairwise intra-tumour heterogeneity increases with sampling distances in patients

**Figure 6.**
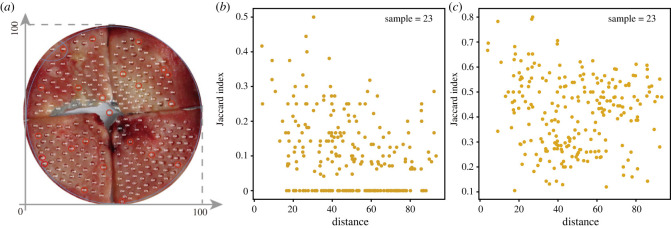
The pairwise Jaccard index between 23 samples with whole exome sequencing data in Ling *et al.* [51]. Panel (*a*) is modified from Ling *et al.* [51], where we normalize sampling distances between 0 and 100. The largest distance 100 refers to the diameter of the whole patient tissue. The 23 biopsy samples used in our analysis are labelled with red circles in Ling *et al.* [51], which are the samples with exome sequencing. (*b,c*), In our simulation, we used sub-clonal mutations, i.e. mutations do not present in all cells, to calculate Jaccard index. Here we filter out the clonal mutations from the data as well. As the tumour purity in real samples seldom reaches to 100%, the clonal mutations might not present in all samples. Here, we show (*b*) the Jaccard distribution after filtering the mutations present in half of all biopsies (Pearson correlation coefficient = −0.309, *p*-value = 1.69 × 10^−6^) and also (*c*) the Jaccard distribution after filtering the mutations present in all biopsies (Pearson correlation coefficient = 0.094, *p*-value = 0.152).

We have demonstrated that the relationship between Jaccard index and sampling distance is qualitatively stable across two- and three-dimensional models as well as different sampling methods. We see a similar pattern in a study of colorectal tumours [60], where the pairwise comparison between samples from the same patient are available through whole exome sequencing data (see their main figure 4). More specifically, multi-region sampling ranging from 4 to 13 samples per tumour were taken for 10 carcinomas. The divergent exonic point mutations (their *x*-axis) between samples increases with physical distance (their *y*-axis), which is consistent with our results. In Ling *et al.* [51], 300 biopsies were collected from a hepatocellular carcinoma, 23 of which are whole exome sequencing samples. We extract the information of sampling distances and exonic mutations from their work, and construct the Jaccard index of these 23 samples pairwisely over their physical distance (see figure 6). In our simulations, we use sub-clonal mutations to calculate Jaccard index, because including clonal mutations shared by all cells does not add any real value but only shrinks the Jaccard index into a small range, thus with a lower resolution to distinguish patterns. Correspondingly, when we calculate the Jaccard index in these patient biopsies, we exclude the clonal mutations as well. While there might be some noise in the real data, where a clonal mutation rarely presents exactly in all samples, we compared two filters where mutations are excluded if they are present in more than half of or in all biopsies. We see a similar pattern with negative correlation between Jaccard index and sampling distance when we filter out the shared mutations in half of the samples.

## 4. Discussion

We developed a computational model that tracked the dynamic movement of each cell and variation divergence, which revealed the relationship of spatial heterogeneity distribution with sampling size and tumour expansion modes. We used push rates to model slow and fast growth modes, where small push rates refer to surface growth and large push rates to exponential expansion without spatial constraints. We implemented two alternative pushing algorithms, where pushing happens in a random direction or towards the nearest empty spot. Furthermore, we recorded the mutation accumulation during all growth modes and applied different sampling methods, i.e. completely random sampling and margin-centre sampling, with various sampling sizes.

Under the surface growth (small push rates), the accumulation of mutations is concentrated in a continuous space, and mutations arising in different original cells can form clear boundaries in space. When the push rate increases, the mutations become more spatially dispersed, which agrees with the conclusion of Chkhaidze *et al.* [34] in simulating driver mutations under the action of pushing. We further showed that small push rates introduce high stochasticity in the system. While the final frequency that an early mutation can reach in small push rates has the same mean expectation compared with large push rates, the variance is much higher. We showed the cumulative VAF distribution follows a linear relationship with 1/frequency for sufficiently high push rates independent of sampling size. We can infer mutation rates based on the method proposed in Williams *et al.* [23]. However, a small sampling size will overestimate the mutation rates compared with the true value in our simulations. When the push rate decreases, the mutation cumulative VAF distributions deviate largely from a linear relation, and such a mutation rate inference becomes less meaningful. This is consistent with Chkhaidze *et al.* [34], where they also showed that the inference of mutation rates becomes less accurate under stronger spatial constraints. While they mainly focused on the patterns of phylogenetic trees under spatial constraints with random punch or needle sampling, we are interested in further quantifying the relation between ITH and sampling distance, which can be served as a baseline expectation of how mutation accumulation diverges when space competition and constraint is the main driving force of the ITH.

While mutation heterogeneity can reveal a tumour’s life history [68] and the patterns of ITH in space are important to understand and improve tumour treatments, systematic studies to quantify those properties are still rare [34,39]. We used the Jaccard index to quantify the mutation heterogeneity between samples and analyse how sampling distance, methods and sizes will impact on the ITH spatial pattern. Our results show that the sampling distance will quickly increase the ITH between samples when push rates are small, which is emphasized if the sampling size is also smaller. On the contrary, high push rates will always maintain a certain level of mutation spatial mixing even with a small sampling size. These results agree with some observations in clinical data. Gates *et al.* [69] sequenced primary glioma biopsy samples at defined distances and recorded Jaccard indices over sample distance. Their data showed that the genetic heterogeneity increased with the sample spatial distance. Similar observations were reported in Cross *et al.* [60], where the number of distinct exonic point mutations increase with the physical sample distance in colorectal tumours. We constructed the Jaccard index distribution of 23 biopsies from [51] over the relative sample distance and further confirmed our conclusion in patient samples. To test the robustness of this observation, we applied margin-centre sampling to compare with completely random sampling. Both the ITH patterns in the centre and margin regions are consistent with our results under completely random sampling, with the centre region revealing a lower ITH compared with the margin region. In addition, our results under three-dimensional simulations remain similar to our two-dimensional results with some quantitative deviations. In a summary, our model gives a baseline expectation of mutation divergence when only space competition is considered, which is a stable pattern independent of the sampling methods and the ecological growth mode of a tumour. Deviation from this may reflect other important mechanisms, e.g. the interactions with tumour microenvironment, which may break the mutation divergence pattern over space.

Our model provides a quantitative analysis of how growth modes, sampling distance and size impact on the measurements of intra-tumour heterogeneity. Those results confirm the importance of obtaining spatial information in understanding tumour evolution, as well as the possible deviation of estimated evolutionary properties such as mutation rates introduced by sampling details.

## Data Availability

All R code and Python code of full pipeline descriptions and settings used for mapping are available from the GitHub repository: https://github.com/SYSU-BioEvoLab/Spatial_Heterogeneity [70]. Supplementary material is available online [71].
